# A Comparative Study on Electro-Optic Effects of Organic N-Benzyl-2-Methyl-4-Nitroaniline and Morpholinium 2-Chloro-4-Nitrobenzoate Doped in Nematic Liquid Crystals E7

**DOI:** 10.3390/polym12122977

**Published:** 2020-12-13

**Authors:** Pravinraj Selvaraj, Karthick Subramani, Che-Ju Hsu, Chi-Yen Huang

**Affiliations:** 1 Department of Physics, National Changhua University of Education, Changhua 500, Taiwan; slpravinraj@gmail.com; 2 Department of Physics, University College of Engineering, BIT-Campus, Anna University, Tiruchirappalli 620 024, India; rvsskarthick@gmail.com; 3 Graduate Institute of Photonics, National Changhua University of Education, Changhua 500, Taiwan

**Keywords:** organic molecule, N-benzyl-2-methyl-4-nitroaniline (BNA), morpholinium 2-chloro-4-nitrobenzoate (M2C4N), liquid crystal, density functional theory

## Abstract

Improvements in electro-optical responses of LC devices by doping organic N-benzyl-2-methyl-4-nitroaniline (BNA) and Morpholinium 2-chloro-4-nitrobenzoate (M2C4N) in nematic liquid crystals (LCs) have been reported in this study. BNA and M2C4N-doped LC cells have the fall time that is fivefold and threefold faster than the pristine LC cell, respectively. The superior performance in fall time of BNA-doped LC cell is attributed to the significant decrements in the rotational viscosity and threshold voltage by 44% and 25%, respectively, and a strong additional restoring force resulted from the spontaneous polarization electric field of BNA. On the other hand, the dielectric anisotropy (Δ*ε*) of LC mixture is increased by 16% and 6%, respectively, with M2C4N and BNA dopants. M2C4N dopant induces a large dielectric anisotropy, because the phenyl-amine/hydroxyl in M2C4N induces a strong intermolecular interaction with LCs. Furthermore, BNA dopant causes a strong absorbance near the wavelength of 400 nm that filters the blue light. The results indicate that M2C4N doping can be used to develop a high Δ*ε* of LC mixture, and BNA doping is appropriate to fabricate a fast response and blue-light filtering LC device. Density Functional Theory calculation also confirms that BNA and M2C4N increase the dipole moment, polarization anisotropy, and hence Δ*ε* of LC mixture.

## 1. Introduction

Nematic liquid crystals (LCs) have been successfully conquered in every corner of our modern world because of their electro-optic applications such as micro-displays [1], flat panel and flexible displays [2,3], and focusing systems [4]. The fast response LC plays a crucial role to improve the motion blur in moving pictures, color contrast, and cross-talk [5,6]. Specifically, the sub-millisecond switching time is required to implement the field sequential color technology of LC displays (LCDs) [7]. Various technologies have been proposed to improve the response time of LCs, such as thin cell gap [8], tuning the optical phase shift [9], an overdrive/undershoot drive scheme [10], new switching modes [11], and using LCs with ultralow viscosity [12]. Some researchers have attempted to accelerate the LC response by incorporating the guest entities, i.e., polymer dispersed liquid crystal (PDLC) and polymer network liquid crystal (PNLC), which are the most efficient routes to achieve the fast response time of LC devices [13,14]. However, the refractive index mismatch of ingredients results in the light scattering, and the polymer structures affect the LC alignment and thus cause incomplete dark and low bright states [15]. Moreover, the required high operation voltage is unfavorable for LCD applications [14]. Likewise, nanoparticle (NP) dispersion in the LC matrix has been considered as a promising technique for achieving fast response time, low power consumption, or bright display colors [16]. Nevertheless, NP doping is still a challenging issue because of the nonuniform dispersion and NP aggregation.

In our previous studies, the rutile titanium dioxide (TiO_2_) NPs with silane-coating had been used to improve the electro-optical properties of nematic LCs with high dielectric anisotropy [17]. Rutile TiO_2_ NPs reduce the threshold and driving voltages due to suppressed screen effect. The fall time of LC cells initially increases and then decreases with the increased TiO_2_ concentrations because of the change in the interaction between the LCs and alignment layer. Furthermore, the doping of organic N-benzyl-2-methyl-4-nitroaniline (BNA) in nematic LCs had been reported for the first time [18]. BNA-doped LC cell had a fall time that was 5× faster than the pristine LC cell. Aiming to LCs with tiny doping, organic BNA doping emerges more remarkable decrement in fall time of the LC device than other dopings, for example, the doping of functionalized silver NPs in LCs induced a turn-off time that was 1.5× faster than the pristine LCs [19]; the doping of silver nanowires in PNLCs resulted in a 2× faster turn-on time than the pristine PNLCs [20]. Afterward, BNA doping was further used to fabricate a fast response large-aperture LC lens with a turn-off time that was∼6 times faster than the LC lens fabricated with the pristine LCs [21]. Consequently, the subsecond level of switching time in the 6mm-aperture LC lens became possible by BNA doping.

Similar to BNA, Morpholinium 2-chloro-4-nitrobenzoate (M2C4N) also has outstanding nonlinear optics (NLO) properties [22] and realizes a high second harmonic generation (SHG) efficiency, which is about 1.43 times higher than urea measured by the Kurtz powder technique [23]. Up to now, the effects of M2C4N in the nematic LCs have not been discussed yet. In the current paper, the electro-optical properties of BNA and M2C4N dopants in nematic LCs are demonstrated and compared. The transmission spectra were used to observe the absorbance of the BNA and M2C4N-doped LC cells. The dielectric spectra and voltage-dependent transmission curves of the BNA and M2C4N-doped LC cells were measured to determine the threshold voltage (*V_th_*) of cell and the dielectric anisotropy (Δ*ε*) and birefringence (Δ*n*) of LC mixture, and the results were used to calculate the splay elastic constant of LC mixture. Notably, M2C4N doping has more enhancement in the Δ*ε* of LC mixture than BNA doping. The phase transition temperatures (*T_NI_*) of the BNA and M2C4N-doped LC cells were observed to confirm the tendency in the order parameter (*S*) of LC mixture. The response times of the BNA and M2C4N-doped LC cells were measured and revealed that the BNA and M2C4N-doped LC cells had the fivefold and threefold faster fall times than the pristine LC cell, respectively. The experimental results conclude that M2C4N doping can be used to develop a high Δ*ε* of LC mixture, and BNA doping is appropriate to fabricate a fast response and blue-light filtering LC device. Moreover, Density Functional Theory (DFT) calculations demonstrate the molecular geometries, polarizability, and dipole moment of the LC mixtures, and further the interactions between the organic dopant and LC molecule.

## 2. Materials and Methods

**Figure 1:**
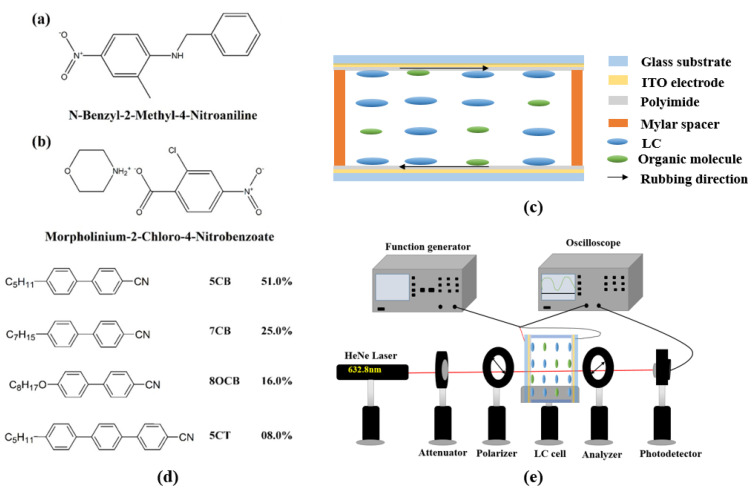
Molecular structures of (**a**) BNA, and (**b**) M2C4N. (**c**) Scheme of cell structure. (**d**) Molecular structure of LC E7. (**e**) Scheme of experimental setup for V-T curve measurement.

Figure 1a shows the molecular structure of organic BNA, which has a melting point of 105 °C. The synthesized process is the same as shown in [18]. M2C4N was synthesized by dissolving 2-Chloro-4-nitrobenzoic acid (2.01 g, 0.01 M) and Morpholine (0.87 g, 0.01 M) with 1:1 equimolar ratio in 30 ml and 10 ml of acetonitrile, respectively. The 2-Chloro-4-nitrobenzoic acid and Morpholine were purchased from Sigma-Aldrich Inc., Bangalore, India. After continuous stirring for 5 h, the Morpholine solution was added dropwise to the 2-Chloro-4-nitrobenzoic acid solution at room temperature. The thoroughly mixed solution became dark yellow in tint with warm heat, and an immediately white precipitate was dispersed (which is insoluble in acetonitrile), and it was separated and dried in a hot air oven at 40 °C. Figure 1b shows the molecular structure of organic M2C4N, which has a melting point of 169.75 °C [22,23].

The commercial 5 ± 0.1 μm-thick empty cell (Heng An Precision Inc., Miaoli City, Taiwan) composed of two indium–tin–oxide (ITO) glass substrates was used in this study. As shown in Figure 1c, the inner surfaces of the substrates were coated with homogeneous (or vertical) polyimide and rubbed in the antiparallel direction. The thickness of the empty cell was confirmed with the interference method. Figure 1d shows the molecular structure of nematic LC E7 (Daily Polymer Corp., Kaohsiung City, Taiwan) used in the experiment. It had a *T_NI_* of 64 °C, Δ*n* of 0.22, rotational viscosity (*γ*) of 232.6 mPas, Δ*ε* of 14.1, and the splay, twist, and bend elastic constants *K*_11_, *K*_22_, and *K*_33_ of 11.1, 5.9, and 17.1 pN, respectively, at 20 °C. In our experiment, the LC mixtures composed of the organic molecule (M2C4N or BNA) and nematic LC E7 with various ratios were stirred ultrasonically for 15 min at room temperature. The concentrations of organic dopants were set to 0, 1, 2, and 3 wt%. Finally, the commercial empty cell was filled in the LC mixture by capillary action. Once the concentration of organic dopant exceeded 3 wt%, the changes in the electro-optical properties of LC cell were almost saturated. By observing the appearance of LC mixture and the polarizing optical microscope (POM) images of LC cell, the organic molecules were well dissolved in LCs without any interface formation between the LCs and organic molecules if the organic molecule concentration was lower than 5 wt%. Once the organic molecule concentration exceeded 5 wt%, the LC mixture became jelly form. Therefore, the organic molecule was considered as a dopant in this experiment.

Optical textures of the LC cells were characterized by using a POM (Microtech POL 3000, M&T optics Co.,Ltd., Taipei, Taiwan) to observe *T_NI_* of LC mixture, where the LC cells were heated from the nematic to isotropic phase at a rate of 0.25 °C/min by using a temperature controller (T95-PE Linkam, Super Chroma Enterprise Ltd., Taipei, Taiwan). The voltage-dependent transmissions (V–T) of the BNA and M2C4N-doped LC cells were measured using the following setup. The LC cell was placed between a pair of crossed polarizers which transmission axes had an angle of 45° with respect to the rubbing direction of cell. A He–Ne laser with a wavelength of 632.8 nm was normally incident on the LC cell, where a square-wave voltage with a frequency of 1 kHz was subjected to the cell, as shown in Figure 1e. The *γ* of the LC mixtures were measured by the transient current method [24]. The pretilt angles of the LC cells were below 3° measured by the crystal rotation method [25]. The Δ*n* of the LC cells were derived from the phase retardation technique [26]. The polar anchoring energies of the LC cells remained at a constant of ~1.1 × 10^−4^ J/m^2^ measured by the high electric field techniques [27]. The dielectric spectra of the LC cells were carried out by LCR meter (Hioki 3532-50, Donho Ltd., Taipei, Taiwan) with an applied alternating current (AC) field of 0.01 V/μm at frequencies from 42 to 5 MHz.

**Figure 2:**
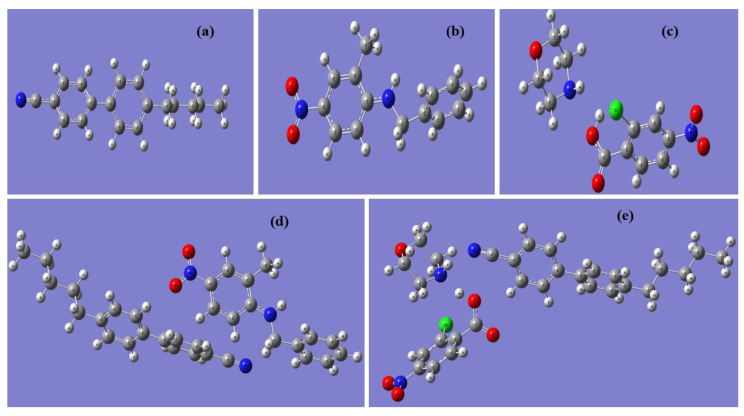
Optimized geometries for (**a**) 5CB, (**b**) BNA, (**c**) M2C4N, (**d**) 5CB + BNA, and (**e**) 5CB + M2C4N by Density Functional Theory (DFT).

As shown in Figure 2, the optimized geometries of 5CB, BNA, M2C4N, 5CB + BNA, and 5CB + M2C4N were performed using DFT with Becke-3-Lee-Yang-Parr (B3LYP) at the 6–31 + G (2d, p) basis sets with the aid of Gaussian ′09 software [28]. The DFT protocol was treated with unrestricted spin orbitals. DFT was also used to qualitatively explain the effects of BNA and MC4N on LC 5CB, because 5CB was the major component of E7, as shown in Figure 1d. Notably, Polarization Continuum Model would be appropriate to quantitatively calculate the organic molecule/LC system. It will be further studied in subsequent works. In Figure 2, the major bond lengths or angles have been discussed as follows. The cyanide (C≡N) bond lengths in 5CB, 5CB + BNA, 5CB + M2C4N are similar at 1.16 Å, indicate the C≡N bond length in 5CB is not affected by the addition of BNA or M2C4N. The amine (N−H) bond lengths in BNA, M2C4N, 5CB + BNA, 5CB + M2C4N are 1.01, 1.02, 1.01, and 1.02 Å, respectively, also indicate the N−H bond lengths in BNA and M2C4N remains the same after doping into 5CB. The hydroxyl (O−H) bond length of M2C4N decreases from 1.02 to 0.90 Å with doping into 5CB. Furthermore, the N−H···N and O−H···N bonds correlate with the charge transfer (CT) between the LC and organic molecule. The N−H···N bond length and angle in 5CB + BNA are 3.82 Å and 84.07°, respectively, and those in 5CB + M2C4N are 2.22 Å and 169.12°. The O−H···N bond length and angle in 5CB + M2C4N are 3.88Å and 136.94°. The parameters such as polarizability (*α*), polarizability anisotropy (Δ*α*), dipole moment (*μ*), absorbance spectra, highest occupied molecular orbital (HOMO), and lowest unoccupied molecular orbital (LUMO) of the LC mixture can be obtained from the optimized geometries. Subsequently, the change in Δ*ε* of the LC mixture can be deduced with Maier-Meier Equations (1)–(3) [29].

(1) $$\varepsilon_{∥}=1+\frac{NFh}{\varepsilon_{0}}\left\{\alpha +\frac{2}{3}\Delta \alpha S+\frac{F\mu^{2}}{3k_{b}T}(1-(1-3\cos^{2}\theta )S\right\},$$

(2) $$\varepsilon_{⊥}=1+\frac{NFh}{\varepsilon_{0}}\left\{\alpha -\frac{1}{3}\Delta \alpha S+\frac{F\mu^{2}}{3k_{b}T}(1+\frac{1}{2}(1-3(\cos^{2}\theta )S\right\},$$

(3) $$\Delta \varepsilon =\frac{NFh}{\varepsilon_{0}}\left\{\Delta \alpha -\frac{F\mu^{2}}{2k_{b}T}(1-3{\left(\cos\theta \right)}^{2})\right\}S,$$

where Δ*ε* is defined as the difference between the permittivity parallel (*ε*_∥_) and perpendicular (*ε*_⊥_) to the molecular axis at 1 kHz; *N* is the molecular number density; *ε*_0_ is the vacuum permittivity; *K_b_* is the Boltzmann constant; *F* and *h* are the reaction field factor and the cavity factor, respectively; *θ* is the dipole moment orientation angle relative to the long principal axis. Consequently, the *α*, Δ*α*, can be defined as follows:

(4) $$\alpha =\frac{\alpha_{xx}+\alpha_{yy}+\alpha_{zz}}{3},$$

(5) $$\Delta \alpha =\alpha_{xx}-\frac{\alpha_{xx}+\alpha_{yy}}{2},$$

where *α_xx_* is the molecular polarizability parallel to the molecular long principal axis and *α_yy_* and *α_zz_* are the molecular polarizabilities perpendicular to the molecular long principal axis.

## 3. Results and Discussion

**Figure 3:**
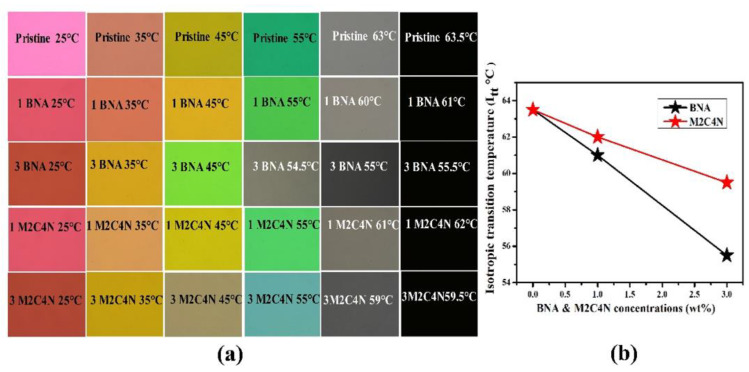
(**a**) Polarizing optical microscope (POM) photographs and (**b**) *T_NI_* of the BNA-LC and M2C4N-LC mixtures.

Figure 3a shows the POM photographs of the BNA and M2C4N-doped LC cells at various temperatures. The rubbing directions of the cells were placed at 45° concerning the transmission axes of the crossed polarizers. The POM photographs show uniform colors throughout the cells, indicate the BNA and M2C4N were well dissolved in the LC matrix without any aggregation and interface formation. The uniform colors in the POM photographs remained unchanged even after several cycles of voltage applications. With increasing temperature, only color shifts were observed in the POM photographs due to the change in the Δ*n* of LC mixture. Figure 3b shows the *T_NI_* of the LC mixtures at various concentrations. Here the aromatic structure of phenyl ligands in the organic dopants has enabled the efficient π-π strong interactions between the host LCs and guest organic molecules, helping to decrease the *T_NI_* of LC mixture [30,31]. Notably, phenyl-amine/methyl (from BNA) and phenyl-amine/hydroxyl/chloride (from M2C4N) substitutes participate in strong intermolecular Coulomb interactions with the cyanide (CN) group of LC molecule [2,32]. As a result, BNA and M2C4N doping decrease the *T_NI_* of LC mixtures by 12% and 6%, respectively. The *T_NI_* of M2C4N-LC mixture is higher than that of BNA-LC mixture, due to the higher melting point by the phenyl-chloride atoms in M2C4N [2,33].

Furthermore, the *T_NI_* of LC mixture is linearly proportional to the *S* and Δ*n*, according to Equations (6) and (7) [34,35].

(6) $$S=(1-\frac{T}{T_{NI}}{)}^{\beta },$$

(7) $$\Delta n=\Delta n_{o}{\left(1-\frac{T}{T_{NI}}\right)}^{\beta },$$

where *T* is ambient temperature, Δ*n_0_* is the birefringence of the LC mixture at 0 K, and *β* is a material parameter. For many of the LC compounds studied, *β* are approximately 0.25 and insensitive to materials [36]. Equation (6) is only valid for *T* sufficiently smaller than *T_NI_* [37]. In this study, *T/T_NI_* is less than 0.5 and thus *S* can be estimated by substituting *T* = 298 K, *β* = 0.25, and *T_NI_* into Equation (6). The phenyl groups of BNA and M2C4N molecules induce a strong intermolecular π-π interaction with the polar substituents of LC molecule so that BNA and M2C4N doping decreases the *T_NI_*, Δ*n,* and *S* of LC mixture [30,31,38], as shown in Table 1. BNA-LC mixture has a lower Δ*n* than the M2C4N-LC mixture, possibly because the two benzene rings of BNA are attached at an angle of 80° that disturbs the alignment of LCs [39]. Because *K*_11_ is proportional to the square of *S* [18], the organic dopants also decrease the *K*_11_ of LC mixture.

**Figure 4:**
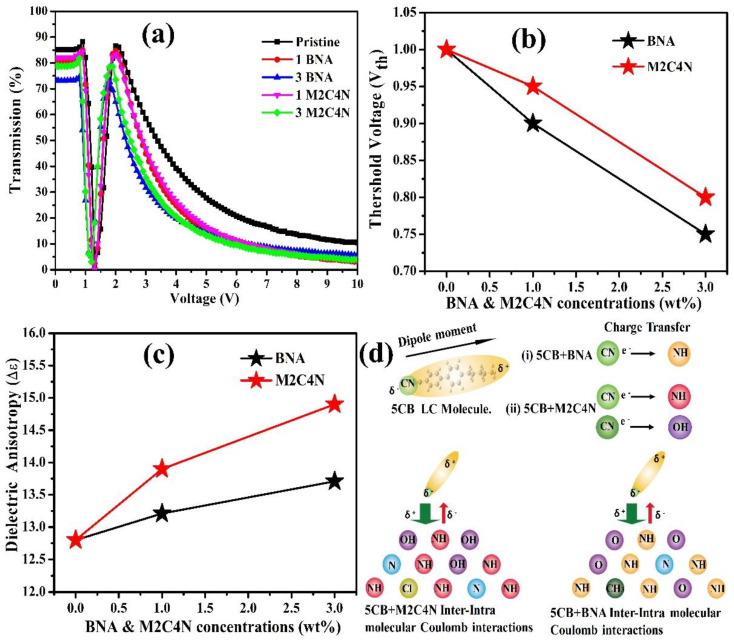
(**a**) V–T curves, (**b**) *V_th_*, (**c**) Δ*ε* as functions of BNA and M2C4N-doped LC cells at 1 kHz, respectively. (**d**) Schematic illustration of charge transfer (CT) effects in the 5CB-M2C4N (Left side) and 5CB-BNA (Right side), respectively.

Figure 4a depicts that the V-T curves of the doping cells at room temperature. The curve shifts toward the low-voltage side with the increasing dopant concentration, representing the organic dopant assists to decrease the operating voltage of LC cell. A decrease in maximum transmission has been obtained. This is because that the organic dopant changes the refractive index of the LC mixture, resulting in a refractive index mismatch between the interfaces or absorption of light by the LC mixture. The transmission change in the V-T curve is related to the varied phase retardation, according to Equation (8):

(8) $$T_{r}=\sin^{2}2\theta \sin^{2}(\delta /2),$$

where *T_r_* is the transmission of the LC cell, *θ* is the angle between the rubbing direction of cell and the transmission axis of polarizer, and *δ* is the phase retardation. If *δ* reaches an even multiple of π, the *T_r_* will be zero, as indicated in Figure 4a. The POM images of the doping cells with various voltages were measured to determine the *V_th_* of the cell [18]. *V_th_* was defined as the voltage at which the color of the POM image began to change, indicating the initial distortion of LCs in the center of the cell. Figure 4b shows that *V_th_* decreases around 25% and 20% for BNA and M2C4N-doped LC cells, respectively, due to the decrement in *K*_11_ and the increment in Δ*ε* of LC mixture by Equation (9) [2].

(9) $$V_{th}=\pi \sqrt{\frac{K_{11}}{\varepsilon_{0}\Delta \varepsilon }}.$$

Moreover, Δ*ε* of LCs mixture increases with the increasing dopant concentration, as shown in Figure 4c. The increased Δ*ε* can be explained by the following ways: (i) a presence of Phenyl rings and a polar terminal group usually causes a larger Δ*ε* [30]. (ii) However, atoms of the major component on the surfaces of BNA and M2C4N change their electronic state attributable to the CT effect. This CT effect is illustrated as a graphical abstract in Figure 4d, where 5CB is the major component of LC E7. In the BNA and M2C4N, the amine (NH) and hydroxyl (OH) atoms have rather large positive charges. On the other hand, the LC 5CB is polarized, resulting in a partial negative charge at the CN group. Consequently, the BNA and M2C4N have a strong intermolecular Coulomb attraction with the CN group of LC 5CB because of large positive charge on the amine and hydroxyl linking groups, which results in an enormous electro-optic effect by giving a stronger perturbation to the LC mixtures [40]. Compared with the BNA dopant, the M2C4N dopant induces a stronger intermolecular interaction with LCs and hence a more increase in the Δ*ε* of LC mixture, owing to the richer aromatic electrophilic substitutions of amine and hydroxyl groups. This strong Coulomb inter-and intra-molecular interactions enhance the *μ, α*, Δ*α*, and hence Δ*ε*, which has been confirmed with the DFT analysis hereafter.

**Figure 5:**
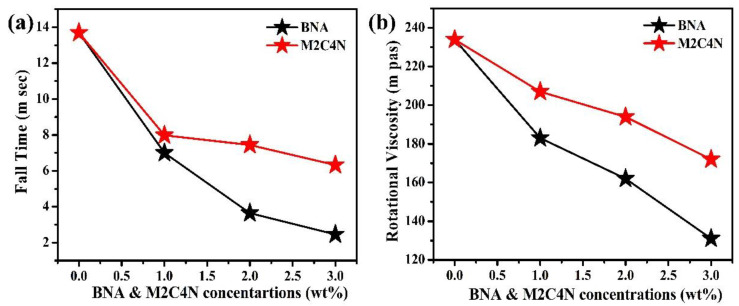
(**a**) *τ_off_* and (**b**) *γ* of BNA and M2C4N-doped LC cells.

Figure 5a shows the response times of the BNA and M2C4N-doped LC cells. Rise (fall) time was defined as the time required for the transmission to change from 90% to 10% (10% to 90%) of the maximum transmission when the cell was turned on from 2 V to 10 V (turned off from 10 V to 2 V). Rise time is significantly smaller than fall time because of the former’s electric torque-driven reorientation, whereas the latter has a free relaxation reorientation. Rise time is almost constant at ~0.68 µs due to the same turn-on voltage. Meanwhile, the fall time of the doping LC cells decreases with the increased dopant concentration because the organic dopant decreases the *γ* of LC mixture and the *V_th_* of cell. The rise time (*τ_on_*) and fall time (*τ_off_*) can be expressed as follows [18]:

(10) $$\tau_{o}=\frac{\gamma d^{2}}{K_{11}\pi^{2}},$$

(11) $$\tau_{on}=\frac{\tau_{o}}{\left|{\left(\frac{V_{app}}{V_{th}}\right)}^{2}-1\right|},$$

(12) $$\tau_{off}=\frac{\tau_{o}}{\left|{\left(\frac{V_{bios}}{V_{th}}\right)}^{2}-1\right|},$$

where *τ_0_* is the relaxation time constant when the LC cell is turned off from *V_app_* slightly higher than *V_th_*, *V_bios_* is the bios voltage, and *d* is the cell thickness. BNA and M2C4N dopants significantly decrease *τ_off_* due to the reduced *V_th_* and *γ*. As shown in Figure 5b, the organic dopants decrease the *γ* of LC mixture because of their phenyl groups. Moreover, the phenyl-alkyl (such as methyl, ethyl, propyl, butyl) group of dopants further decreases the *γ* of LC mixture. Compared with M2C4N-LC mixture, BNA-LC mixture has phenyl-methyl groups so that the more decrease in the *γ* of LC mixture [2,32,33]. An additional restoring force by the spontaneous polarization electric field (SPEF) of the organic dopant also decreases the *τ_off_* of cells. If we consider the polar organic molecule as a dipole. The direction of the resultant dipole moment surrounding the organic dopant (local regions) could be different from the director of LCs [41,42]. When no electric field is applied to the cell, the LCs near the local region orient along the resultant dipole moment direction, but other LCs still align parallel to the cell substrate. The amplitude of local electric field *E* induced by the resultant dipole moment *μ* can be written as [42]

(13) $$E=\frac{\mu }{4\pi \varepsilon r^{3}}{\left(1+3\cos^{2}\theta \right)}^{1/2},$$

where *r* represents the radial radius, *θ* represents the polar angle, and *ε* represents the dielectric permittivity of the medium. As a sufficiently high electric field is applied to the cell, the LCs near the local regions as well as other regions reorient parallel to the applied electric field. Once the applied field is turned off, the LCs neat the local regions tend to rapidly rewind back to their previously resultant dipole moment directions, and hence decrease the fall time of the cell further. Table 2 shows the *μ* of BNA is larger than that of M2C4N, indicating BNA dopant induces a stronger SPEF than M2C4N. Consequently, BNA doping causes the more decrease in the *τ_off_* of cell than M2C4N doping, due to the more decreases in the *V_th_* and *γ* and the stronger additional restoring force by the SPEF of dopant. As shown in Table 2, the increments in *μ*, *α*, and Δ*α* were more significant than the decrement in *S* of LC mixture with the addition of organic molecule, indicating that the increased Δ*ε* was mainly attributed to the increments in *μ*, *α*, and Δ*α* according to Equations (1)–(3).

**Figure 6:**
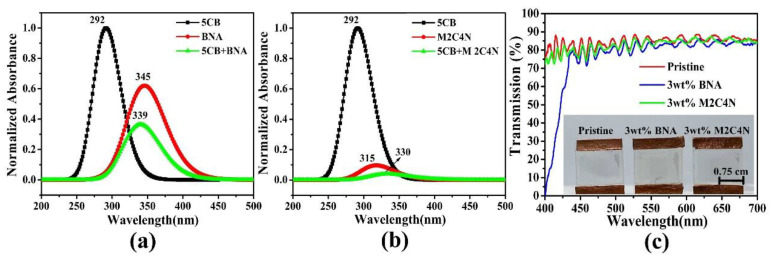
(**a**)Theoretical absorption spectra for 5CB, BNA, 5CB + BNA, (**b**)5CB, M2C4N, and 5CB + M2C4N by using DFT. (**c**) Transmission spectra of the BNA and M2C4N-doped LC cells. Inset shows the sample photos of the pristine LC E7, 3 wt% BNA-doped, and 3 wt% M2C4N-doped LC cells under daylight illumination.

Figure 6a,b show the normalized absorbance peaks for 5CB, BNA, 5CB + BNA, M2C4N, and 5CB + M2C4N appear at 292 (π → π *), 345, 339, 315, and 330 nm (*n* → π *), respectively, due to the presence of nitro, amine, and methyl groups [43,44]. By correlating the optimized geometries (Figure 2), the BNA dopant shifts the absorbance peak of the LCs toward the long-wavelength side, due to the amine group in BNA has a strong intermolecular interaction with the CN group in LCs. Similarly, the M2C4N dopant shifts the absorbance peak of the LCs toward the long-wavelength side because the primary amine and hydroxyl groups in M2C4N strongly connect with the CN group in LCs [44]. The organic dopants shift the absorbance peaks of LC mixture toward long wavelengths, indicating they decrease the HOMO-LUMO energy bandgap (Δ*E*) of LC mixture, owing to the inverse relationship between Δ*E* and wavelength [18,45,46,47]. Figure 6c shows the measured transmission spectra of the pristine, 3 wt% BNA-doped, and 3 wt% M2C4N-doped LC cells over the visible range. The tiny loss is because that the organic dopant changes the refraction index of the LC mixture, resulting in the refractive index mismatch between the glass substrate and LC layer. The enormous light loss near 400 nm in the BNA-doped LC cell originates from the strong absorbance of BNA molecules [48]. The inset of Figure 6c shows the sample photos of the LC cells, the yellow tint is related to the used light source and the BNA absorbance at the wavelength of 400 nm. These results indicate that BNA-doped LC cell plays the role of blue light filter in LC devices, thereby retina is protected from some harmful effects.

## 4. Conclusions

The electro-optical properties of BNA and M2C4N-doped LC cells have been successfully demonstrated in this study. BNA doping has the more decrease in the *T_NI_* of LC mixture than M2C4N doping owing to the phenyl-methyl in BNA. As summarized in Table 3, the *V_th_* of the BNA and M2C4N-doped LC cells are decreased by 25% and 20%, respectively, because the organic dopants decrease the *K*_11_ and increase the Δ*ε* of LC mixture. M2C4N doping causes a more increase in the Δ*ε* of LC mixture than BNA doping, because the phenyl-amine/hydroxyl in M2C4N induces a stronger interaction with the CN group of LCs. The 3 wt% BNA and M2C4N-doped LC cells have the fall time that is fivefold and threefold faster than the pristine one, respectively. The faster response of BNA-doped LC cell is attributed to the more decreases in the *V_th_* and *γ* of LC mixture and the stronger additional restoring force by the BNA’s SPEF. The DFT calculations reveal that organic dopants increase the *E_int_*, *μ, α,* and Δ*α*, and decrease the Δ*E* so that increase the Δ*ε* of LC mixture. BNA doping has a drastic absorbance at ~400 nm that enables to act as a blue light intensity filter in LC devices. The comparitive study indicates that M2C4N doping can be used to develop a high Δ*ε* of LC mixture and BNA doping is appropriate to develop a fast response and blue-light filtering LC device. Furthermore, the organic dopants have strong absorbances for ultraviolet (UV) light, which is potential to anti-UV applications, such as anti-UV smart windows and glasses.

## Figures and Tables

**Table 1:** Concentration-dependent *T_NI_*, *S*, Δ*n*, and *K*_11_ of BNA-LC and M2C4N-LC mixtures.

Sample	(*T_NI_* °C)	*S*	Δ*n*	*K*_11_ (pN)
Pristine LC	63.5	0.582	0.219	11.49
1 wt% BNA-LC mixture	61.0	0.573	0.191	9.60
3 wt% BNA-LC mixture	55.5	0.552	0.190	6.92
1 wt% M2C4N-LC mixture	62.0	0.577	0.210	11.26
3 wt% M2C4N-LC mixture	59.5	0.568	0.195	8.56

**Table 2:** Calculated *μ*, *α*, Δ*α,* and Δ*E* of 5CB, BNA, 5CB + BNA, M2C4N, and 5CB + M2C4N molecules via Gaussian 09-DFT methods.

Molecular Geometry	*μ* (Debye)	*α* (a.u.)	Δα (a.u.)	Δ*E* (eV)
5CB	06.30	238.29	215.16	4.66
BNA	08.80	207.99	163.50	3.79
5CB + BNA	14.07	446.15	339.01	3.91
M2C4N	05.49	181.70	23.60	4.01
5CB + M2C4N	12.90	447.09	376.10	3.80

**Table 3:** Summary of BNA and M2C4N dopants.

	Electro-Optic Effect of Doping Cell				Coulomb Interaction with CN Group of LCs	**Absorbance Peak of Dopant**	**Absorbance Peak of Dopant 5CB Mixture**
	Decrease in *V_th_*	Increase in Δ*ε*	Decrease in *γ*	Decrease in *τ_off_*			
BNA	25%	6%	44%	5×	Phenyl-amine/methyl	345 nm	339
M2C4N	20%	16%	25%	3×	Phenyl-amine/hydroxyl/chloride	315 nm	330
